# Reboxetine Plus Oxybutynin for OSA Treatment: A 1-Week, Randomized, Placebo-Controlled, Double-Blind Crossover Trial

**DOI:** 10.1016/j.chest.2021.08.080

**Published:** 2021-09-20

**Authors:** Elisa Perger, Luigi Taranto Montemurro, Debora Rosa, Stefano Vicini, Mariapaola Marconi, Lucia Zanotti, Paolo Meriggi, Ali Azarbarzin, Scott A. Sands, Andrew Wellman, Carolina Lombardi, Gianfranco Parati

**Affiliations:** a Istituto Auxologico Italiano, IRCCS, Sleep Disorders Center & Department of Cardiovascular, Neural and Metabolic Sciences, San Luca Hospital, Milan, Italy; b Department of Medicine and Surgery, University of Milano-Bicocca, Milan, Italy; c Division of Sleep and Circadian Disorders, Brigham and Women’s Hospital and Harvard Medical School, Boston, MA; d IRCCS Fondazione Don Carlo Gnocchi, Milan, Italy

**Keywords:** antimuscarinic and norepinephrine reuptake inhibitors, OSA, pharmacologic treatment, upper airway, vigilance

## Abstract

**Background:**

The recent discovery that a combination of noradrenergic and antimuscarinic drugs improved upper airway muscle function during sleep and reduced OSA severity has revitalized interest in pharmacologic therapies for OSA.

**Research Question:**

Would 1 week of reboxetine plus oxybutynin (Reb-Oxy) be effective on OSA severity?

**Study Design and Methods:**

A randomized, placebo-controlled, double-blind, crossover trial was performed comparing 4 mg reboxetine plus 5 mg oxybutynin (Reb-Oxy) vs placebo in patients with OSA. After a baseline in-laboratory polysomnogram (PSG), patients underwent PSGs after 7 nights of Reb-Oxy and 7 nights of placebo to compare apnea-hypopnea index (AHI), which was the primary outcome. Response rate was based on the percentage of subjects with a ≥ 50% reduction in AHI from baseline. Secondary outcomes included Epworth Sleepiness Scale (ESS) score and psychomotor vigilance test (PVT) values. Home oximetry evaluated overnight oxygen desaturation index (ODI) throughout treatment.

**Results:**

Sixteen subjects aged 57 [51-61] years (median [interquartile range]) with a BMI of 30 [26-36] kg/m^2^ completed the study. Reb-Oxy lowered AHI from 49 [35-57] events per hour at baseline to 18 [13-21] events per hour (59% median reduction) compared with 39 [29-48] events per hour (6% median reduction) with placebo (*P* < .001). Response rate for Reb-Oxy was 81% vs 13% for placebo (*P* < .001). Although ESS scores were not significantly lowered, PVT median reaction time decreased from 250 [239-312] ms at baseline to 223 [172-244] ms on Reb-Oxy vs 264 [217-284] ms on placebo (*P* < .001). Home oximetry illustrated acute and sustained improvement in the oxygen desaturation index on Reb-Oxy vs placebo.

**Interpretation:**

The administration of Reb-Oxy greatly decreased OSA severity and increased vigilance. These results highlight potential possibilities for pharmacologic treatment of OSA.

**Clinical Trial Registration:**

ClinicalTrials.gov; No.: NCT04449133; URL: www.clinicaltrials.gov

## Graphical Abstract

**Figure:**
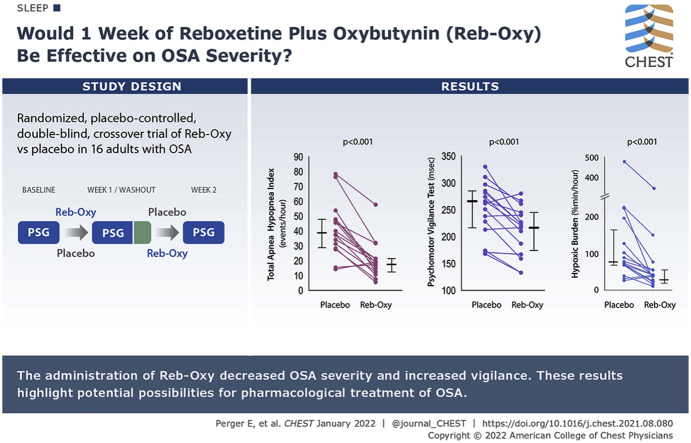

**Take-home Points**

**Study Question:** Will the Reb-Oxy combination be effective in reducing OSA severity?

**Results:** The Reb-Oxy combination reduced the AHI by 59% from baseline together with an increase in oxygen saturation during the night. Moreover, Reb-Oxy improved sustained vigilance compared with placebo and did not cause severe adverse events.

**Interpretation:** CPAP is still the most common treatment for patients with OSA, and although it is often effective, many patients find it intolerable and therefore remain untreated. Our results provide strong evidence supporting OSA pharmacologic therapy.

OSA is one of the most common sleep disorders and affects approximately 10% to 17% of the general population.^1^ Due to repetitive collapse of the pharyngeal airway during sleep, OSA leads to intermittent oxygen desaturations, sleep fragmentation, excessive daytime sleepiness, and cardiovascular impairment, and long-term OSA is associated with increased morbidity and mortality.^2^^,^^3^

Although OSA is effectively alleviated with the use of CPAP and, for some individuals, oral appliances or multimodal approaches,^4^ these treatments are often poorly tolerated by patients, and compliance is relatively low.^5^ Efforts to develop pharmacologic therapies for the treatment of OSA have accelerated over the last two decades, but currently no pharmacologic intervention has been approved for clinical use.

One of the key pathophysiological traits in OSA is the loss of upper airway (UA) dilator muscle activity at sleep onset and the lack of reactivation (muscle compensation) during sleep in response to UA obstruction. Research in animals improved the understanding of the state-dependent neurotransmitters involved in pharyngeal muscle activation during sleep, providing evidence that both noradrenergic and antimuscarinic processes are involved. Specifically, the impairment of noradrenergic activity is now thought to play a key role in the sleep-related hypotonia of pharyngeal muscles, mostly during non-rapid eye movement (NREM)^6^, ^7^, ^8^ sleep, and muscarinic activity is primarily involved in rapid eye movement (REM) muscle atonia.^9^^,^^10^

It has been shown that a fixed-dose combination of atomoxetine (a noradrenergic agent) and oxybutynin (an antimuscarinic compound)^11^ reduced the frequency of obstructive events (apnea-hypopnea index [AHI]) by 63% and improved genioglossus responsiveness to negative pressure swings approximately threefold in 20 patients with OSA over a single night of treatment. In a study of healthy individuals, a combination of reboxetine (a noradrenergic agent) and hyoscine butylbromide (an antimuscarinic drug) improved activity of the tensor palatini muscle and UA resistance during NREM sleep.^12^ Preliminary data also suggested an improvement in OSA severity of approximately 35% following a single night of reboxetine plus hyoscine butylbromide.^13^ Given that the putative mechanism of action of this combination is the stimulation of the hypoglossal motor pool in the brainstem, we considered that a combination of reboxetine plus oxybutynin (Reb-Oxy) might have greater therapeutic potential than reboxetine plus hyoscine, as oxybutynin can reach a higher concentration in the brainstem parenchyma.^14^ Because these processes have been identified only recently, no attempts to stimulate the pharyngeal muscles with these drugs for longer than a single night have yet been made.

Therefore, in the current study, we performed a randomized, placebo-controlled, double-blind, crossover trial to evaluate the efficacy of the Reb-Oxy combination on the severity of OSA following 1 week of treatment. We hypothesized that Reb-Oxy would reduce AHI (primary outcome) and oxygen desaturation indexes from baseline to a greater extent than with placebo. Moreover, we investigated the effects of this combination on OSA pathophysiological (or endotypic) traits,^15^^,^^16^ also exploring whether patients’ baseline characteristics could predict the treatment success.

## Patients and Methods

### Patients

Both male and female patients between 18 and 70 years of age with a recent (< 1 year) diagnosis of OSA were eligible for study enrollment. Subjects treated with CPAP were included in the study (Table 1) only if they exhibited poor compliance (use of CPAP < 4 h per night for 70% of nights), and they were asked to completely stop the treatment at least 2 weeks prior to the baseline PSG. Exclusion criteria included any clinically significant neurologic, psychiatric, or cardiovascular disorder; untreated narrow angle glaucoma; hypertension requiring more than three drugs to be controlled; use of respiratory stimulants or depressants, hypnotics, CNS stimulants, or other medicaments known to interact with study drugs; central sleep apnea; pregnancy; or history of benign prostatic hyperplasia or urinary retention that may be exacerbated by antimuscarinic medications.

**Table 1:** General Characteristics of the Study Population

Characteristic	Value
Age, y	57 [51-61]
Male sex	14 (87.5)
Height, cm	180 [171-184]
Weight, kg	94 [77-105]
BMI, kg/m^2^	30 [26-36]
Waist circumference, cm	116 [103-123]
Neck circumference, cm	43 [39-46]
Mallampati score (1/2/3/4)	1 (6.3)/10 (62.5)/4 (25)/1 (6.3)
Tonsils score (1/2/3/4)	15 (93.7)/1 (6.3)/0/0
Smoke	8 (50)
Previous OSA treatment	
C-PAP	5 (31.2)
Comorbidities	
Hypertension	7 (44)
Diabetes	1 (6.3)
Dyslipidemia	7 (44)
Hypothyroidism	3 (18.8)
Rheumatoid arthritis	1 (5.6)
Medications	
ACE inhibitor/ARB	6 (35)
CCB	1 (6.3)
Diuretics	1 (6.3)
Antilipidemics	4 (25)
Antidiabetics	1 (6.3)
Antithrombotics	2 (12.6)

Data are presented as median [interquartile range] or No. (%). ACE = angiotensin-converting enzyme; ARB = angiotensin receptor blocker; CCB = calcium channel blocker.

Participants were enrolled from July 2020 to October 2020 through our sleep clinic (Istituto Auxologico Italiano) following a prescreening evaluation for inclusion and exclusion criteria based on the clinical history. The trial ended when the previously calculated sample size was reached.

The study was approved by the Ethics Committee and by the Italian drug agency AIFA (Agenzia Italiana del Farmaco). Informed consent in writing was obtained from all study participants. The study was registered at ClinicalTrials.gov.^17^

### Study Design

This was a randomized, double-blind, placebo-controlled, cross-over, Phase II, single-center efficacy study of the Reb-Oxy combination in adults with OSA.

Study participants underwent further eligibility screening with 1 night in-laboratory baseline PSG (Embla), which served as the baseline for AHI and other PSG end points. Participants were eligible for randomization if AHI at baseline PSG was > 15 events per hour. Eligible participants were then randomized equally to first receive 4 mg reboxetine plus 5 mg oxybutynin (Reb-Oxy) or matching placebo (2 capsules). Subjects started taking study drug at home the day after the baseline PSG immediately prior to bedtime for 7 days. A 7- to 10-day washout preceded the switch to the other arm of the study. During the entire at-home period (6 nights on placebo and 6 nights on Reb-Oxy), the patients underwent full night pulse oximetry testing (Nonin Medical Inc.). On the final night of dosing for each arm, participants underwent an in-laboratory PSG to evaluate OSA severity. The predefined primary outcome variable was the change in AHI from baseline. Secondary outcomes were: response rate based on ≥ 50% reduction in AHI; proportion of participants with AHI < 15 events per hour; change in subjective sleepiness with Epworth Sleepiness Scale (ESS) and psychomotor vigilance test (PVT); and change from baseline in PSG parameters (oxygen desaturation index [ODI] at 3% threshold and hypoxic burden). Karolinska Sleepiness Scale (KSS), Patient Global Impression of OSA-Severity (PGI-S), arousal index, periodic limb movement index, and descriptive summary of nightly change with at-home pulse oximetry (ODI 4%) were also assessed.

### Randomization and Blinding

Study medications were prepared by ST Pharma PRO SRL and were placed in identical capsules that could not be identified by study personnel or participants. Participants were randomly assigned in a 1:1 equal allocation ratio to receive the active treatment dose or placebo first using a blocked randomization (block size of 2). Each participant was assigned a unique number (randomization number) that encoded the participant’s assignment to one of the two arms of the study. The randomization list was produced and validated by a statistician not involved in patient recruitment and external to the hospital. No stratification was expected for any characteristics. Subjects, care providers, investigators, and outcomes assessors were blinded to the treatment allocation (quadruple blinding). Study treatment was dispensed the morning after PSG screening. Once all data analyses were completed and reviewed, the database was locked and the intervention allocations were unblinded for statistical analysis

### Data Analyses and Measurements of Outcomes

Overnight PSG recordings and scoring were performed in accordance with the American Academy of Sleep Medicine rules.^18^ All studies were scored by the same specialized sleep clinician, blinded to treatment assignment, according to American Academy of Sleep Medicine criteria.^19^ AHI, ODI 3%, arousal index, and periodic limb movement index were calculated from the PSG. The OSA-specific hypoxic burden (respiratory event-related oxygen desaturation area under pre-event plus baseline curve, per hour) was also calculated.^20^^,^^21^ ODI at 4% threshold level (ODI 4%) was collected during at-home pulse oximetry for each night of treatment. Adverse events were recorded at each visit.

Pathophysiological traits causing sleep apnea were (endotypes) estimated during NREM sleep using established automated methods and executed by using custom software (Endo-Phenotyping Using Polysomnography; MATLAB, MathWorks).^15^^,^^16^^,^^22^ Details are provided in e-Appendix 1.

The ESS questionnaire was taken to evaluate subjective somnolence over the preceding week of treatment,^23^ and the KSS was taken to measure the situational sleepiness in the late afternoon prior to the in-laboratory PSG. The PGI-S was used to rate the participant’s impression of disease severity. A validated 3-min PVT evaluated the sustained-attention and reaction time (RT) by measuring the speed with which subjects responded to a visual stimulus.^24^^,^^25^ The median RT, the number of lapses (defined as RT > 500 ms; ie, inability to respond in a timely fashion when a stimulus was present), and the reciprocal RT as a measure of speed (1/RT) (lapses included) were studied. The aforementioned evaluations together with respiratory rate, ECG, and three measurements of BP were performed without coffee intake in the previous 3 h and at the same time of the day prior to the PSG.

### Statistical Analysis

Individuals were enrolled until 16 patients completed baseline and both treatment nights. The study was powered to detect an AHI reduction with Reb-Oxy (percent reduction from baseline) by 50+/-50% more than placebo (alpha 5%, power 80%); SD of the effect was estimated from a previous trial.^11^

Data are presented as median [interquartile range]. Continuous variables were compared by using a two-tailed Wilcoxon matched-pairs signed-rank test. Categorical data were analyzed by using Fisher exact test. A linear mixed effect model for AHI (percent reduction from baseline) accounting for treatments, period, and sequence as fixed effects and subjects as a random effect is included in e-Appendix 1.

For the endotypic traits, the effect of the Reb-Oxy combination and placebo vs baseline were also modeled by using a linear mixed effects model, with treatments as fixed effects and subjects as a random effect. Additional details are provided in e-Appendix 1.

To evaluate the predictors of response to Reb-Oxy from baseline characteristics, we performed a univariate linear regression analysis, including baseline age, BMI, PSG characteristics (AHI, ODI, fraction of events that were hypopneas, and mean desaturation associated with an event), and each endotype as independent variables. The percent change in AHI was the dependent variable. Associations were exploratory and were not adjusted for multiple comparisons.

A *P* value < .05 was considered statistically significant. Statistical analyses were performed by using GraphPad Prism 6.0 (McKiev Software) and MATHLAB (MathWorks).

## Results

### Subjects

**Figure 1:**
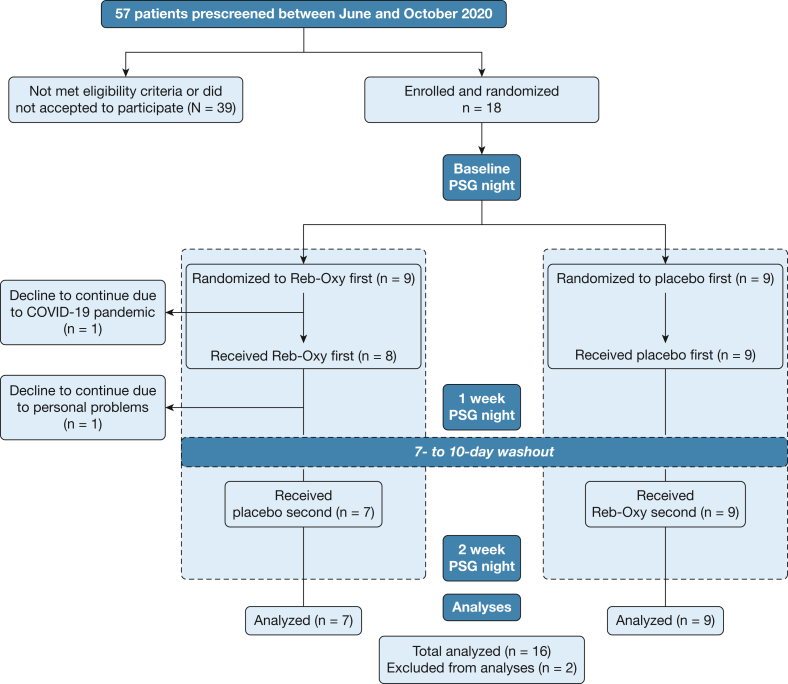
Consolidated Standards of Reporting Trials diagram of the clinical trial. PSG = *polysomnogram*; Reb-Oxy = reboxetine plus oxybutynin.

Eighteen subjects were enrolled in the study and underwent a baseline PSG night; all individuals were eligible for randomization based on AHI > 15 events per hour (Consolidated Standards of Reporting Trials diagram in Fig 1). One subject dropped out prior to starting the first treatment period (during the second wave of COVID-19 in Milan, Italy; active drug period). One subject dropped out at the end of the first treatment period (also active drug period) as the patient was unable to continue (personal problems).

Data from 16 participants were available for analysis of OSA severity at baseline and on both nights following the week of drug or placebo intake. Table 1 presents the characteristics of these subjects. None had a history of UA surgery.

The following side effects were reported during the study on the Reb-Oxy night: urinary hesitation (difficulties in initiating micturition in the morning, n = 7 male subjects); dry mouth during the night and in the morning (n = 10); sexual dysfunction (erectile dysfunction in the morning or decreased libido, n = 3 male subjects); and brief sensation of palpitation (n = 1) and insomnia symptoms (difficulty initiating and maintaining sleep, n = 1). On placebo, chest pain (n = 1) and side pain (n = 1) were observed (e-Table 1). No participants experienced severe side effects or severe adverse events in either arm. No differences were found in terms of resting BP, heart rate, or ECG among the visits.

The results relative to primary and secondary outcomes were upheld when adjusted for sequence and period effects in a linear mixed effect model analysis (e-Fig 1, e-Tables 2 and 3). A significant sequence effect was found in the analysis of AHI percent reduction. Adjusted results showed a reduction of the placebo effect, suggesting a possible mild carryover effect on placebo when it was administered after Reb-Oxy (e-Appendix 1 provides a detailed model). Secondary outcomes such as hypoxic burden or PVT were not affected by period or sequence.

### Effect of Reb-Oxy on AHI, Oxygen Saturation, and Sleep Architecture

**Figure 2:**
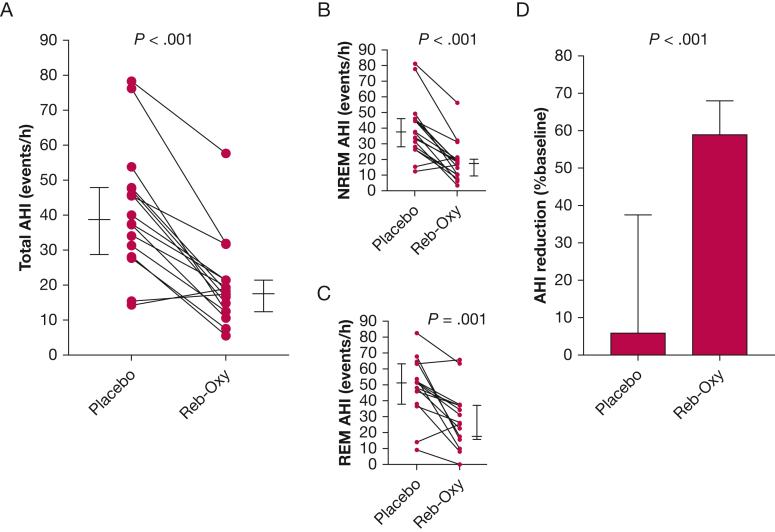
Individual data showing the effect of Reb-Oxy on total AHI (A) and during NREM (B) or REM (C) sleep stages. Longer horizontal lines indicate median values, and shorter lines indicate 25th and 75th percentiles. D, Group data showing percentage of AHI changes from baseline on placebo and on Reb-Oxy. AHI = apnea-hypopnea index; NREM = non-rapid eye movement; Reb-Oxy = reboxetine plus oxybutynin; REM = rapid eye movement.

**Figure 3:**
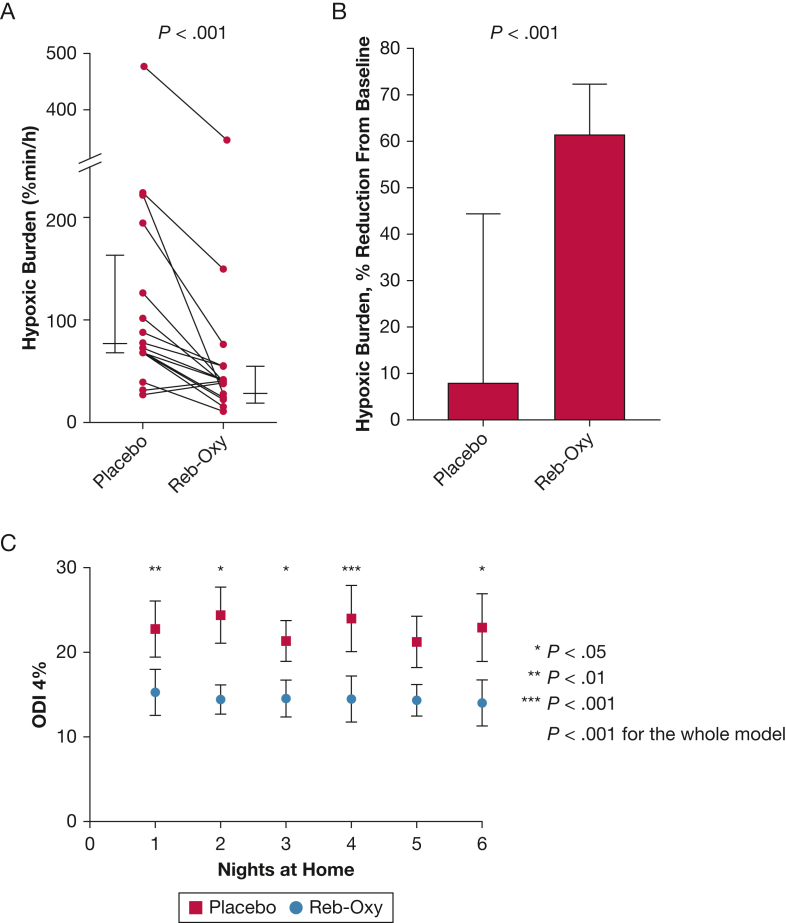
A-C, Effect of Reb-Oxy on desaturation index. A, Hypoxic burden as individual data. Longer horizontal lines indicate median values, and shorter lines indicate 25th and 75th percentiles. B, Group data showing percentage of hypoxic burden changes from baseline on placebo and on Reb-Oxy. C, Analysis of repeated measures of ODI 4% obtained during at-home pulse oximetry during placebo (red squares) and during Reb-Oxy (blue dots) weeks. Data were compared by using a mixed effect model including treatment, time, and time × treatment interaction as fixed effects and subjects as a random effect. Only treatment effect was significantly associated with ODI 4% (dependent variable). P values for day-by-day multiple comparisons between placebo and Reb-Oxy arms are adjusted by using the Šidák method. AHI = apnea-hypopnea index; NREM = non-rapid eye movement; ODI = oxygen desaturation index; Reb-Oxy = reboxetine plus oxybutynin.

Reb-Oxy reduced the AHI by a median of 26 events per hour, or 59% (expressed as the median value of all reductions), compared with baseline and by 20 events per hour, or 59% compared with placebo (Fig 2, Table 2). The vast majority of patients (81%) experienced a reduction in AHI > 50% on the treatment night, and 37% of the patients on Reb-Oxy had an AHI < 15 events per hour. Effects of the intervention on AHI specific to REM and NREM sleep stages, hypoxic burden, ODI, arousal index, and sleep architecture are shown in Table 2. Reb-Oxy significantly reduced hypoxic burden and ODI (*P* < .001 and *P* = .021, respectively). Considering that a hypoxic burden > 53% min/h has been previously associated with higher cardiovascular-related mortality,^20^ Reb-Oxy reduced the hypoxic burden below this threshold in 69% of the study sample. Individual data on hypoxic burden are reported in Figures 3A and 3B. Reb-oxy significantly reduced the number of arousals compared with baseline and placebo, and sleep architecture was unchanged with the exception of a trend for reduced REM sleep and increased N2 sleep on Reb-Oxy compared with placebo. No difference in periodic leg movements were observed among the three nights.

**Table 2:** OSA Severity and Sleep Architecture Baseline on Placebo and on Drug Combination for All Participants (N = 16)

Variable	Baseline	Placebo	Reb-Oxy	*P* Value
AHI total, events/h	48.7 [34.8 to 56.6]	38.7 [29.0 to 47.8]	18.0 [12.5 to 21.4]	< .001
% Change from baseline		5.9 [–4.5 to 37.5]	59.2 [53.3 to 68.1]	< .001
AHI supine, events/h	60.4 [52.7 to 81.9]	56.3 [44.9 to 76.0]	33.7 [25.3 to 48.1]	< .001
% Change from baseline		7.0 [0.4 to 27.2]	51.1 [30.9 to 64.3]	< .001
Proportions of patients with AHI reduction > 50% from baseline		13%	81%	< .001
Proportion of patients with AHI < 15 events/h		6%	37%	.080
Hypoxic burden, % min/h	90.8 [69.5 to 154]	75.5 [68.1 to 168.0]	39.7 [25.4 to 55.3]	< .001
% Change from baseline		7.7 [–17.3 to 44.5]	61.5 [38.2 to 72.5]	< .001
ODI 3%, events/h	42.7 [32.3 to 53.0]	36.8 [23.8 to 43.2]	31.4 [19.1 to 37.7]	.021
% Change from baseline		11.1 [–4.6 to 25.3]	29.0 [13.3 to 42.6]	.025
ODI 4%, events/h	34.8 [23.9 to 43.9]	30.1 [17.4 to 40.0]	20.1 [13.3 to 28.2]	.001
% Change from baseline		7.7 [–7.7 to 38.2]	38.5 [21.1 to 49.7]	.016
Arousal index, events/h	30.6 [20.7 to 47.7]	26.6 [14.1 to 34.7]	10.7 [7.6 to 16.8]	.003
Total sleep time, min	329.5 [301.0 to 368.8]	323.5 [274.4 to 351.4]	321.8 [283.0 to 362.9]	.376
Sleep efficiency, % TIB	71.2 [59.9 to 76.2]	71.7 [60.8 to 83.5]	69.7 [64.0 to 73.3]	.504
N1, % TST	3.7 [2.4 to 7.3]	3.5 [2.8 to 4.5]	5.4 [2.7 to 9.9]	.102
N2, % TST	63.5 [55.3 to 68.1]	62.9 [58.5 to 68.7]	68.0 [58.4 to 75.8]	.051
N3, % TST	16.2 [10.9 to 22.1]	17.4 [9.5 to 26.3]	15.9 [6.8 to 23.0]	.117
REM, % TST	18.1 [13.8 to 21.4]	16.2 [13.2 to 17.9]	10.2 [5.1 to 15.5]	.057
PLM index, events/h	0.0 [0.0 to 2.8]	0.0 [0.0 to 2.8]	0.5 [0.0 to 2.8]	.457
Heart rate, beats/min	78 [71 to 90]	82 [72 to 93]	79 [69 to 87]	.700
Systolic BP, mm Hg	133 [124 to 145]	126 [118 to 135]	120 [115 to 138]	.234
Diastolic BP, mm Hg	82 [75 to 89]	84 [75 to 92]	80 [73 to 88]	.065

Data are presented as median (interquartile range); % changes are expressed as the median of the group percent change. *P* values compare placebo vs Reb-Oxy. AHI = apnea–hypopnea index; N1-2-3 = non-rapid eye movement stage 1-2-3; ODI = oxygen desaturation index; PLM = periodic leg movements; Reb-Oxy = reboxetine plus oxybutynin; REM = rapid eye movement sleep; TIB = time in bed; TST = total sleep time.

### Effect of Reb-Oxy on ODI at Home

ODI 4% obtained during at-home pulse oximetry was collected on average (SD) 5.7 (0.8) nights on Reb-Oxy and 5.4 (1.0) nights on placebo. Group results are shown in Figure 3C for each night. In the mixed effects model, only treatment (Reb-Oxy vs placebo) was associated with a significant change in ODI 4% (*P* < .001); there were no effects related to time or to the interaction between time and treatment.

### Effect of Reb-Oxy on Subjective Questionnaires and Vigilance

**Figure 4:**
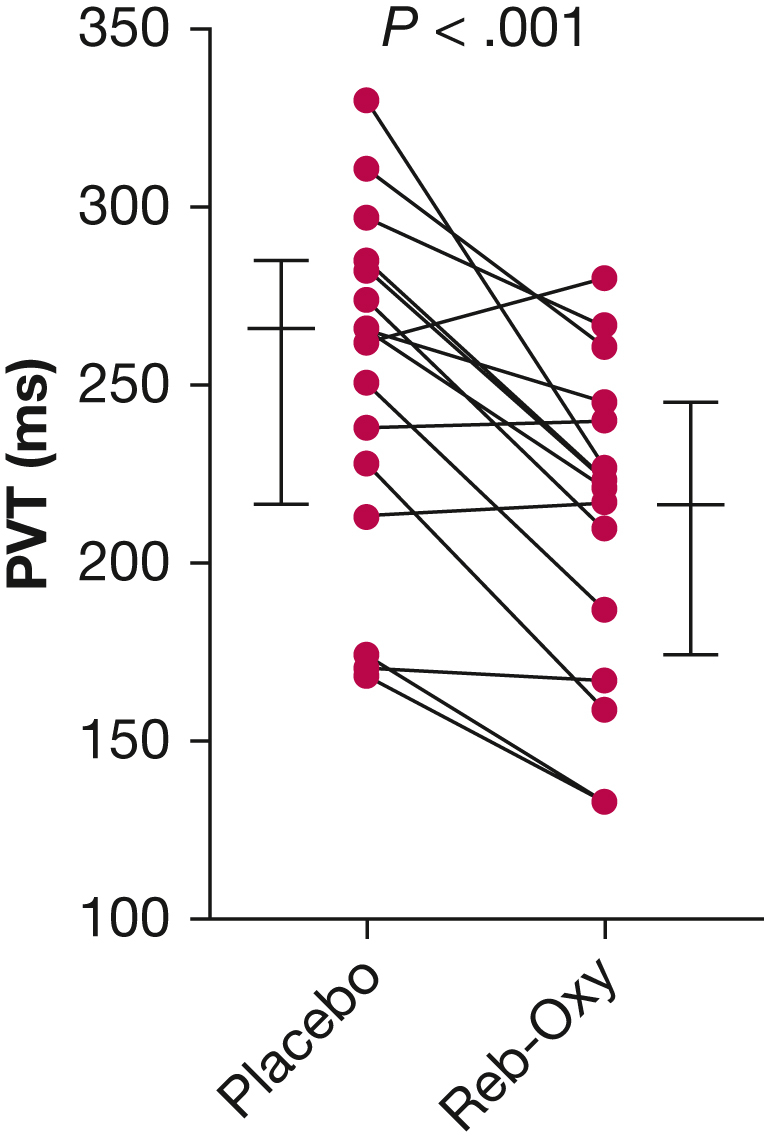
Effect of Reb-Oxy on PVT reaction time. Longer horizontal lines indicate median values, and shorter lines indicate 25th and 75th percentiles. PVT = psychomotor vigilance test; Reb-Oxy = reboxetine plus oxybutynin.

Reb-Oxy did not significantly improve subjective indexes related to sleepiness, impression of disease severity, or vigilance when considering group data (Table 3). Regarding subjective sleepiness, four of five patients with an ESS score > 6 at baseline experienced improvement in the score, from 11 [3-12.5] to 6 [1.5-6.5], although this did not reach statistical significance (*P* = .19). PGI-S improved on Reb-Oxy compared with baseline, but again this difference did not reach statistical significance (*P* = .087). Despite KSS revealing no change in subjective alertness between treatments, PVT as RT and 1/RT performance significantly improved on Reb-Oxy compared with placebo (Fig 4).

**Table 3:** Results of Questionnaires Regarding Subjective Indexes Related to Sleepiness and Impression of Disease Severity and Objective Vigilance Test (N = 16)

Variable	Baseline	Placebo	Reb-Oxy	*P* Value
ESS	5.0 [4.3 to 9.3]	5.0 [3.0 to 6.0]	5.0 [3.0 to 7.5]	.75
% Change from baseline		0 [–15 to 30]	25 [–10 to 42]	.45
KSS	2.0 [1.0 to 2.8]	1.5 [1.0 to 3.0]	2.0 [1.0 to 2.8]	.53
% Change from baseline		0 [–75 to 25]	0 [–100 to 54]	.75
PGI-S	7.0 [4.0 to 8.0]	4.0 [3.0 to 7.8]	3.5 [2.3 to 6.5]	.184
% Change from baseline		0 [–7 to 33]	21 [–14 to 56]	.59
PVT, reaction time, ms	250 [239 to 312]	264 [217 to 284]	223 [172 to 244]	< .001
% Change from baseline		5 [–7 to 11]	19 [6 to 30]	.02
PVT, lapses	2 (1.0%)	0	3 (1.6%)	.33
PVT, 1/RT	4.0 [3.33 to 4.17]	3.8 [3.5 to 4.5]	4.5 [4.1 to 5.7]	.02

Data are presented as median [interquartile range]; % changes are expressed as the median of the group percent change. *P* values compare placebo vs Reb-Oxy. 1/RT = reciprocal reaction time as a measure of speed; ESS = Epworth Sleepiness Scale; KSS = Karolinska Sleepiness Scale; PGI-S = Patient Global Impression of OSA-Severity; PVT = psychomotor vigilance test; Reb-Oxy = reboxetine plus oxybutynin; RT = reaction time.

### Effect of Reb-Oxy on Pathophysiological Traits

Group data from the mixed effects model of endotypic traits at baseline, on placebo, and on Reb-Oxy are shown in Table 4. Compared with placebo, Reb-Oxy increased muscle compensation by 30% of normal/eupneic ventilatory drive (eupnea), supporting the effect of this combination on UA muscle responsiveness. However, Reb-Oxy reduced the arousal threshold by 27% of eupnea (ie, patients woke more easily on active treatment). Vactive was increased on Reb-Oxy by 20% of eupnea compared with baseline but not compared with placebo. No changes were found in loop gain (ie, ventilatory control sensitivity) or Vpassive (ie, passive pharyngeal tissue collapsibility).

**Table 4:** Mixed Effects Model for Effect of Reb-Oxy vs Placebo on Vpassive, Vactive, Muscle Compensation, Arousal Threshold, and Loop Gain During NREM Sleep

Variable	Vpassive (% Eupnea)	Vactive (% Eupnea)	Muscle Compensation (% Eupnea)	Arousal Threshold (% Eupnea)	Loop Gain (Unitless)
Intercept (baseline)	82 [58 to 106]	76 [46 to 107]	–57 [–115 to 1]	139 [107 to 171]	0.60 [0.47 to 0.74]
Placebo vs baseline	6 [–12 to 23] *P* = .52	20 [0 to 41] *P* = .049	11 [–6 to 27] *P* = .198	5 [–16 to 25] *P* = .645	–0.01 [–0.13 to 0.11] *P* = .879
Reb-Oxy vs baseline	17 [–4 to 38] *P* = .11	35 [10 to 60] *P* = .007	40 [17 to 63] *P* < .001	–23 [–43 to –2] *P* = .033	–0.09 [–0.21 to 0.03] *P* = .15
Reb-Oxy vs placebo	11 [–9 to 32] *P* = .259	15 [–9 to 39] *P* = .219	30 [7 to 53] *P* = .012	–27 [–48 to –7] *P* = .01	–0.08 [–0.21 to 0.04] *P* = .192

Data are presented as mean [95% CI]. Values for Vpassive do not represent observed data but rather the underlying collapsibility derived from a sigmoidal transformation function to handle the ceiling effects previously described for these types of data.^16^ Values for Muscle Compensation were calculated from Vactive adjusting for Vpassive such that the effect shown is the additional effect on ventilation above Vpassive (thus representing pharyngeal compensation). NREM = non-rapid eye movement; Reb-Oxy = reboxetine plus oxybutynin.

### Predictors From Patients’ Baseline Characteristics

An inverse relationship was found between the change in AHI and baseline mean desaturation, expressed as the average difference between the highest and lowest saturation value during respiratory events: the lower the desaturation, the higher the AHI reduction (*r* = –0.68; *P* = .004). It was also found that the lower the arousal threshold, the higher the AHI reduction (*r* = –0.56; *P* = .024). A direct relationship was noted between baseline Vpassive and AHI reduction: the higher the Vpassive (better airway anatomy), the greater the AHI reduction (*r* = 0.5; *P* = .047).

## Discussion

This study provides experimental evidence that Reb-Oxy administered prior to bedtime substantially reduces OSA severity (AHI) after 1 week of treatment. In addition to the AHI reduction, Reb-Oxy also exerted a significant effect on indexes of hypoxemia such as ODI and hypoxic burden. Reb-Oxy also improved performance on the vigilance testing. OSA alleviation was likely mediated by improved UA muscle activity and responsiveness, as suggested by the approximately 30% increase in muscle compensation on the drugs. Home pulse oximetry recordings showed that Reb-Oxy was effective at improving nocturnal oxygen saturation as early as the first day of treatment, likely due to reduced OSA severity, and its efficacy was maintained through day 7, as shown in the in-laboratory PSG.

Reboxetine is a norepinephrine reuptake inhibitor approved outside the United States for the treatment of major depression. Oxybutynin is an antispasmodic drug that inhibits the muscarinic action of acetylcholine on smooth muscle and is indicated for the treatment of symptoms of bladder instability in patients with neurogenic bladder.

To date, only one published study reported a significant improvement in OSA severity through pharmacologic therapy with noradrenergic and antimuscarinic agents. In their project, Taranto-Montemurro et al^11^ evaluated a combination of atomoxetine 80 mg and oxybutynin 5 mg (ato-oxy) in a single-night study. The ato-oxy combination reduced the AHI from 31 events per hour on placebo to 8 events per hour on active treatment (*P* < .0001). In our study, we performed a baseline PSG on the night prior to starting the drugs and tested again following 1 week of administration, rather than only acutely. The current study therefore extends knowledge by providing evidence for effectiveness of the combination of noradrenergic and antimuscarinic agents administered over a full week. Moreover, we also evaluated subjective and objective responses to the drugs administered. Our population presented low ESS scores, which might have influenced the Reb-Oxy effect on sleepiness. Although patients did not report subjective improvement in sleepiness as a group, patients with higher ESS scores at baseline showed a clinically meaningful improvement with the combination compared with placebo, and there was evidence of improvement in vigilance (PVT reaction time and speed) following 1 week of treatment with Reb-Oxy. However, our study did not show a difference in PVT lapses, suggesting the absence of clinically significant baseline impairment of vigilance in the study population. The improvements in the PVT values may be due to both an improvement of OSA and to the stimulant effects of reboxetine.

Regarding the pathophysiology traits, our finding that Reb-Oxy improved muscle compensation (40% eupnea improvement on drugs vs baseline and 30% vs placebo) is consistent with previous single-night effects of ato-oxy (improved compensation by approximately 29% eupnea). The reduction in arousal threshold we observed is consistent with prior studies. It has indeed been shown in previous experiments that the arousal threshold can be modified by treatment: while the exposure to intermittent hypoxia increases the arousal threshold,^26^ treatment with CPAP reduces the arousal threshold in patients with OSA.^27^ Other potential explanations include the possibility of a stimulant effect from reboxetine or a bias related to the methodology used to measure the arousal threshold^22^: spontaneous arousals occurring during mild flow limitation may have contributed to a low arousal threshold score.

Unlike with ato-oxy, a reduction in loop gain was not observed in the current study. In terms of predictors, responders to ato-oxy^28^ exhibited several signs of reduced collapsibility (lower AHI, higher Vpassive, and higher fraction of hypopneas over total events). Likewise, in the current study, several surrogates of milder collapsibility (higher Vpassive, lower arousal threshold, and lesser mean desaturation) were associated with greater responses to Reb-Oxy, confirming the notion that pharmacologic therapy for OSA may be most efficacious in patients with less severe pharyngeal compromise. The similar findings in the ato-oxy studies and the current Reb-Oxy study is not surprising, as both norepinephrine reuptake inhibitors have a comparable receptor affinity profile, with some differences being that reboxetine has a longer plasma half-life than atomoxetine (approximately 12 h vs approximately 5 h, respectively) and that reboxetine’s major path of elimination is cytochrome P450 (CYP) 3A4 isozymes,^29^ whereas atomoxetine’s is CYP-2D6. It is therefore unlikely that reboxetine causes clinically significant interactions common to other antidepressants.^30^

It has been recently shown by Lim et al^12^ that reboxetine plus hyoscine butylbromide (an antimuscarinic drug) during sleep increased the activity of the tensor palatini muscle, a representative tonic UA muscle, and improved UA resistance in 10 healthy subjects. This combination of drugs also reduced REM sleep and increased N2 sleep compared with placebo, with no effect on sleep efficiency. Although we observed the same trend of REM reduction and increased N2 sleep, the differences in sleep stage distribution between the nights of placebo and Reb-Oxy in our study were not significant. Due to the presence of moderate to severe OSA, the study population probably had an altered sleep quality already that was not further affected by drug intake. Moreover, we administered the medications for 7 days rather than 1 day, and it was previously shown that the negative effects of reboxetine alone on sleep quality tend to disappear with prolonged therapy.^31^

The combined effects of reboxetine plus hyoscine butylbromide was also evaluated in a randomized controlled trial in 12 subjects with OSA.^13^ The combination of these drugs reduced the AHI by approximately 35%, increased the proportion of N2 sleep compared with placebo, and reduced REM sleep. Compared with oxybutynin, hyoscine butylbromide has a low permeability for the blood-brain barrier, possibly resulting in less efficacy.^14^ Concerning safety, previous studies using reboxetine plus hyoscine butylbromide^12^ and ato-oxy^11^ did not show major side effects. Accordingly, we observed no severe major adverse effects, even following 7 days of Reb-Oxy administration.

The true efficacy of Reb-Oxy vs placebo might be underestimated in our study because of the crossover design. We found that the effect observed for placebo was greater when it was administered after Reb-Oxy (sequence effect) (e-Appendix 1), suggesting that a longer washout period should be considered in future crossover trials.

Although our results report the efficacy of Reb-Oxy on OSA severity, our study is a proof-of-concept trial in a limited number of subjects. Larger and longer trials need to be performed to confirm the efficacy and the safety of these drugs in a broad range of subjects with OSA. Moreover, the safety of Reb-Oxy in subjects with cardiac comorbidities also needs to be carefully studied. Considering the short duration of the trial, and to reduce participation burden, we decided to avoid a second baseline PSG. Although Reb-Oxy reduced AHI, hypoxic burden, and arousal index, its impact on subjective sleep quality was not statistically significant in this small trial. Given that the combination showed a trend for reduction in sleepiness in patients with an ESS score > 6, future studies in sleepier patients should be considered.

## Interpretation

The current study showed for the first time that repeated doses of the combination of noradrenergic and antimuscarinic drugs is efficacious for the alleviation of OSA. Specifically, over 1 week, Reb-Oxy provided a 59% reduction in AHI and halved OSA severity in 81% of individuals. Acute effects exhibited on the first night were sustained at the end of the week. Although subjective sleepiness was not reduced in this population, objective PVT results showed promising signs of improvement with no major safety issues. These results provide strong pilot data for the design of larger and longer studies testing these drugs as a pharmacologic therapy for patients with OSA.

## Supplementary Data

e-Online Data
