# Supplementary material for: Reboxetine Plus Oxybutynin for OSA Treatment: A 1-Week, Randomized, Placebo-Controlled, Double-Blind Crossover Trial
Source: Chest. 2021 Sep 20;161(1):237–47. doi: 10.1016/j.chest.2021.08.080 (PMC10835052; doi:10.1016/j.chest.2021.08.080)
Supplement: e-Online Data [file mmc1.pdf]

CHEST Online Supplement

Reboxetine Plus Oxybutynin for OSA Treatment

A 1-Week, Randomized, Placebo-Controlled, Double-Blind Crossover Trial

Elisa Perger, MD; Luigi Taranto Montemurro, MD; Debora Rosa, PhD; Stefano Vicini, MD; Mariapaola Marconi; Lucia Zanotti, PhD; Paolo Meriggi, PhD; Ali Azarbarzin, PhD; Scott A. Sands, PhD; Andrew Wellman, MD, PhD; Carolina Lombardi, MD, PhD; and Gianfranco Parati, MD, PhD

CHEST 2022; 161(1):237-247

Online supplements are not copyedited prior to posting and the author(s) take full responsibility for the accuracy of all data.

© 2021 AMERICAN COLLEGE OF CHEST PHYSICIANS. Reproduction of this article is prohibited without written permission from the American College of Chest Physicians. See online for more details. **DOI:** 10.1016/j.chest.2021.08.080

e-Appendix 1.

Pathophysiological traits causing sleep apnea

Briefly, each trait is defined by spontaneous fluctuations in ventilation (from nasal pressure, mean-normalized) and ventilatory drive (intended ventilation estimated using a chemoreflex model and least-squares regression). Collapsibility was based on the median ventilation during sleep at normal/eupneic ventilatory drive (Vpassive); lower values of Vpassive indicate greater collapsibility[1]. Compensation, the increase in ventilation with rising ventilatory drive, was determined by calculating Vactive (ventilation when ventilatory drive is at the arousal threshold); greater Vactive, for any given Vpassive, reflects greater dilator muscle compensation. Loop gain (LG1, ventilatory control sensitivity) was determined from the ventilatory drive response to reduced ventilation; higher values represent a greater ventilatory control instability. Arousal threshold was measured as the ventilatory drive preceding each scored arousal[2]; low values reflect greater arousability.

Statistical analyses: Effects on Vpassive (collapsibility) were modelled by using a sigmoidal transformation function (slope of 1 at Vpassive = 50%) to handle the known floor and ceiling effects[1] ; changes in collapsibility using our method are only linearly related to underlying collapsibility in the flow-limited range between Vpassive = 0% (apnea) and Vpassive = 100% (open airway). Effects on muscle compensation were estimated by modelling Vactive (same sigmoidal function) while adjusting for Vpassive. Effects on LG and arousal threshold were modelled by using simple linear models.

The effects of placebo and reb-oxy on repeated measures of ODI 4% at home were analyzed using a mixed effects model testing treatment, time, and the interaction between treatment and time as fixed effects and subjects as random effects. Comparisons between ODI on reb-oxy *vs.* placebo at individual time points (days 1-6) were corrected for multiplicity using the Sidak method.

Online supplements are not copyedited prior to posting and the author(s) take full responsibility for the accuracy of all data.

Supplemental results

e-Table 1: Adverse Events (AE) during the week on placebo and the week on reboxetine plus oxybutynin (reb-oxy). None reported severe AE. Comparisons were performed using a Chi-squared test (n = 16).

	**Placebo**	**Reb-oxy**	**p-value**
**Dry mouth**	0	10	<0.01
**Urinary hesitation**	0	7	0.03
**Sexual disfunction**	0	3	0.69
**Palpitation**	0	1	0.31
**Insomnia**	0	1	0.31
**Chest pain**	1	0	0.31
**Side pain**	1	0	0.31
**Headache**	1	0	0.31
**Cramps**	1	0	0.31
**Total n of patients reporting AE:**	2	13	<0.01

Definition of abbreviations: reb–oxy = reboxetine plus oxybutynin;
Data are presented as number (percentage).

e-Table 2. Linear mixed effect model for apnea hypopnea index (AHI, percent reduction from baseline)

**Fixed effects:**	**Mean [95% CI]**	**P-value**
Placebo	11% [-2 to 24]	0.09
Reboxetine + Oxybutynin<br>*Change from Placebo*	+48% [31.5 to 64]	<0.001

Online supplements are not copyedited prior to posting and the author(s) take full responsibility for the accuracy of all data.

e-Table 3. Linear mixed effect model for apnea hypopnea index (AHI, percent reduction from baseline) accounting for sequence and period

**Fixed effects:**	**Mean [95% CI]**	**P-value**
Placebo	1% [-12 to 14]	0.87
Reboxetine + Oxybutynin<br>*Change from Placebo*	+46.5% [31 to 62]	<0.001
Period	+10% [-6.5 to 25.5]	0.2
Sequence	+25% [8 to 41]	0.005

Periods in the model were represented by the following values: -0.5 for Visit 1, 0.5 for Visit 2. Sequences in the model were represented by the following values 0: Placebo first, Reboxy second; 1: Reboxy first, placebo second.

The significant sequence effect suggests that there was a trend for an increased %reduction in AHI from baseline on placebo when the active treatment was administered first (sequence 1, carry-over effect). In order to explore this possibility, we analyzed the patients separately, based on treatment sequence. Figure S1 shows a significant difference between placebo and active treatment in the %reduction of AHI after dividing the patients according to treatment sequence (top graphs). Bottom graphs show a significant increase in %reduction from baseline on the placebo arm on sequence 1 vs sequence 0, but no significant difference between sequences in the Reb4-Oxy4 arm. No period or sequence effects were found in the analysis of the other outcomes (change in AHI [absolute value], ODI 3 and 4%, hypoxic burden, ESS, PVT, KSS, PGI-s, arousal index and periodic leg movements).

Online supplements are not copyedited prior to posting and the author(s) take full responsibility for the accuracy of all data.

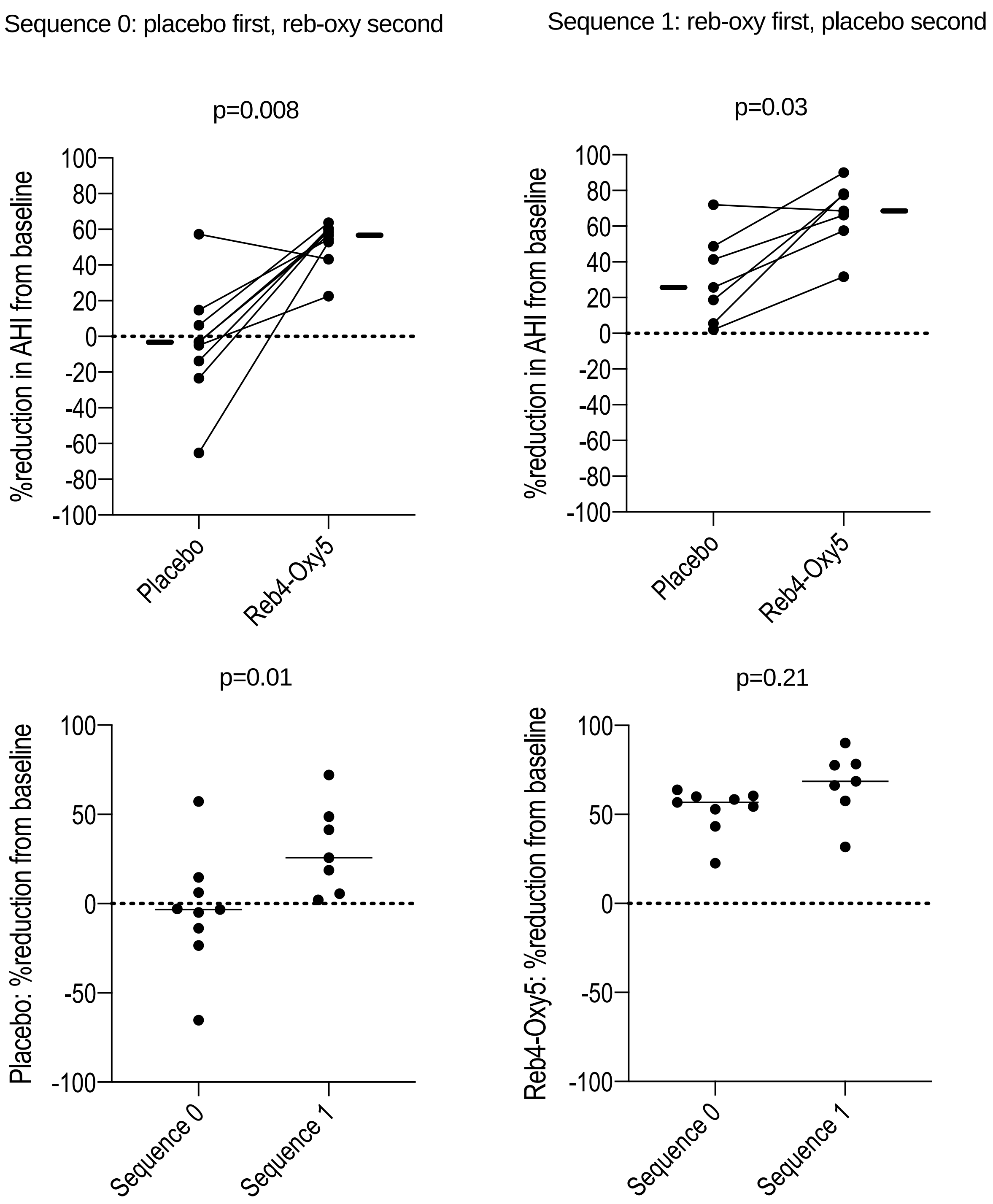

e-Figure 1. Lines indicate medians; sequence 0: placebo first, then reb4-oxy5; sequence 1: reb4-oxy5 first, then placebo.

Online supplements are not copyedited prior to posting and the author(s) take full responsibility for the accuracy of all data.

Supplemental References

S1. Sands SA, Edwards BA, Terrill PI, Taranto-Montemurro L, Azarbarzin A, Marques M, Hess LB, White DP, Wellman A. Phenotyping Pharyngeal Pathophysiology using Polysomnography in Patients with Obstructive Sleep Apnea. *Am J Respir Crit Care Med* 2018: 197(9): 1187-1197.

S2. Sands SA, Terrill PI, Edwards BA, Taranto Montemurro L, Azarbarzin A, Marques M, de Melo CM, Loring SH, Butler JP, White DP, Wellman A. Quantifying the Arousal Threshold Using Polysomnography in Obstructive Sleep Apnea. *Sleep* 2018: 41(1).

Online supplements are not copyedited prior to posting and the author(s) take full responsibility for the accuracy of all data.

CHEST® Online Supplement

Online supplements are not copyedited prior to posting and the author(s) take full responsibility for the accuracy of all data.
